# Supplementary material for: Outdoor Absolute Humidity Predicts the Start of Norovirus GII Epidemics
Source: Microbiol Spectr. 2023 Feb 14;11(2):e02433-22. doi: 10.1128/spectrum.02433-22 (PMC10100787; doi:10.1128/spectrum.02433-22)
Supplement: Supplemental file 1 — Tables S1 to S6. Download spectrum.02433-22-s0001.pdf, PDF file, 0.1 MB [file spectrum.02433-22-s0001.pdf]

Supplementary tables I-VI

Supplementary table I. Weekly lagged correlations for norovirus GII detection rates and absolute humidity across seven seasons.

Absolute humidity (weekly mean) Lag week	Fixed effects F	Coefficient	*p* value
0	0.3	0.02	0.6
1	25	-0.15	7×10^{-7}
2	11	-0.09	0.001
3	1	-0.03	0.2
4	12	-0.1	0.001
5	7	-0.08	0.007
6	2	-0.04	0.2
7	0.3	-0.02	0.6
8	1	-0.03	0.3

Supplementary table II. Weekly lagged correlations for norovirus GII detection rates and temperature across seven seasons.

Temperature (weekly mean) Lag week	Fixed effects F	Coefficient	*p* value
0	0.7	0.011	0.4
1	25	-0.056	7×10^{-7}
2	4	-0.025	0.04
3	5	-0.027	0.03
4	21	-0.050	0.000005
5	8	-0.032	0.005
6	5	-0.027	0.02
7	2	-0.015	0.2
8	2	-0.017	0.2

Supplementary table III. Weekly lagged correlations for norovirus GII detection rates and relative humidity across seven seasons.

Relative humidity (weekly mean) Lag week	Fixed effects F	Coefficient	*p* value
0	3	0.01	0.08
1	1	-0.006	0.3
2	3	-0.008	0.09
3	0.6	0.004	0.5
4	0.03	0.001	0.9
5	0.2	0.002	0.7
6	0.7	0.006	0.3
7	2.7	0.005	0.3
8	2	0.008	0.1
10	0.06	0.001	0.8
12	7	0.02	0.008
14	1	0.006	0.3

Supplementary table IV. Weekly lagged correlations for rotavirus detection rates and absolute humidity across seven seasons.

Absolute humidity (weekly mean) Lag week	Fixed effects F	Coefficient	*p* value
0	7	-0.1	0.007
1	16	-0.1	0.00008
2	13	-0.1	0.0003
3	15	-0.1	0.0001
4	50	-0.3	9×10^{-12}
5	45	-0.2	9×10^{-11}
6	97	-0.3	0.0000….
7	87	-0.3	0.0000….
8	60	-0.3	1×10^{-13}
9	44	-0.2	1×10^{-10}
10	64	-0.3	2×10^{-14}

Supplementary table V. Weekly lagged correlations for rotavirus detection rates and temperature across seven seasons.

Temperature (weekly mean) Lag week	Fixed effects F	Coefficient	*p* value
0	2	-0.02	0.2
1	11	-0.05	0.001
2	13	-0.1	0.0003
3	15	-0.1	0.0001
4	50	-0.3	9×10^{-12}
5	26	-0.06	5×10^{-7}
6	40	-0.07	6×10^{-10}
7	61	-0.09	5×10^{-14}
8	58	-0.08	3×10^{-13}

Supplementary table VI. Weekly lagged correlations for rotavirus detection rates and relative humidity across seven seasons.

Relative humidity (weekly mean) Lag week	Fixed effects F	Coefficient	*p* value
0	3.4	-0.01	0.07
1	0.7	-0.006	0.4
2	2.7	-0.01	0.1
3	0.5	-0.005	0.5
4	0.6	-0.006	0.4
5	0.2	0.003	0.6
6	2	-0.01	0.2
7	0.5	0.006	0.5
8	0.2	0.003	0.7
10	0.09	-0.002	0.8
12	4	0.02	0.04
14	10	0.03	0.002
16	3	0.02	0.09
